# Prognostic Relevance of Changes in Exercise Test Variables in Pulmonary Arterial Hypertension

**DOI:** 10.1371/journal.pone.0072013

**Published:** 2013-09-05

**Authors:** Herman Groepenhoff, Anton Vonk-Noordegraaf, Mariëlle C. van de Veerdonk, Anco Boonstra, Nico Westerhof, Harm J. Bogaard

**Affiliations:** 1 Department of Pulmonology, Institute for Cardiovascular Research, VU University Medical Center, Amsterdam, The Netherlands; 2 Department of Physiology, Institute for Cardiovascular Research, VU University Medical Center, Amsterdam, The Netherlands; Keio University School of Medicine, Japan

## Abstract

**Introduction:**

Exercise variables determined in patients with pulmonary arterial hypertension (PAH) at the time of diagnosis, predict survival. It is unknown whether upon treatment, subsequent *changes* in these exercise variables reflect improvements in survival. The aim of this study was to determine changes in exercise variables in PAH patients and to relate these changes to survival.

**Methods:**

Baseline cardiopulmonary exercise test (CPET) variables and six-minute-walk-distance (6MWD) were available from 65 idiopathic PAH patients (50 females; mean age 45±2yrs). The same variables were determined after treatment (13months) in a sub group of 43 patients. To estimate the association between changes in exercise variables and changes in cardiac function, right-ventricle ejection fraction (RVEF) was measured by cardiac MRI at baseline and after treatment in 34 patients. Mean follow-up time after the second CPET was 53 (range: 4-111) months. Kaplan-Meier analysis was used to relate survival to baseline and treatment-associated changes in exercise variables.

**Results:**

Survivors showed a significantly greater change in maximal oxygen uptake than non-survivors and this change in aerobic capacity was significantly related to changes in RVEF. From baseline until the end of the study period, two patients underwent a lung transplantation and 19 patients died. Survival analysis showed that baseline 6MWD (*p*<0.0001), maximal heart rate (*p*<0.0001) and the slope relating ventilation with carbon dioxide production (*p*≤0.05) were significant predictors of survival, whereas baseline oxygen uptake and oxygen pulse held no predictive value. Treatment associated changes in 6MWD (*p*<0.01), maximal heart rate (p<0.05), oxygen uptake (*p*<0.001) and oxygen pulse predicted survival (*p*<0.05), whereas changes in the slope relating ventilation with carbon dioxide production did not.

**Conclusion:**

Exercise variables with prognostic significance when determined at baseline, retain their prognostic relevance after treatment. However, when changes in exercise variables upon treatment are considered, a different set of variables provides prognostic information.

## Introduction

Patients with pulmonary arterial hypertension (PAH) show specific patterns of gas exchange abnormalities during a cardio pulmonary exercise test (CPET) [1]. For that reason, international guidelines promote CPET to determine exercise capacity and prognosis in PAH [2,3]. Baseline exercise variables affected by pulmonary hypertension and predictive of survival reflect an impaired cardio-circulatory function and a decreased ventilatory efficiency [4–6]: a low six minute walk distance (6MWD), a low maximal oxygen uptake (VO_2_), a low oxygen pulse (O_2_pulse), a high linear regression slope relating ventilation to carbon dioxide production (VE/VCO_2_) and a low maximal heart rate (HR) [4–6].

It was recently reported that in PAH, important prognostic information is provided by treatment induced changes in several established predictors of outcome [7]. That changes in 6MWD are predictive of subsequent survival has been reported in several clinical PAH trials which used exercise time and 6MWD as clinical end-points [8–10]. In addition, a changed exercise induced shunt, as determined by CPET, has been shown to predict outcome in PAH patients [11]. Together, these studies suggest that changes in exercise responses over time, either resulting from disease progression or from a response to PAH specific therapy, are predictive of subsequent survival. However, specific knowledge of the prognostic significance of changes in several CPET variables is lacking. To determine the relevance to prognosis of treatment associated changes in exercise variables, we analyzed the results of CPET and 6MWD at baseline and after PAH specific treatment in a cohort of patients with idiopathic and familial PAH.

## Methods

### Ethics statement

The study protocol was approved by the VU University Medical Centre Institutional Review Board and informed consent was waived due to the retrospective nature of this study.

### Patients

All idiopathic and heritable PAH patients referred to the VU University Medical Centre in Amsterdam between December 2000 till December 2012 who underwent CPET and six minute walk test were included in this retrospective analysis.

At the time of diagnosis, hemodynamic variables were measured in all patients by a diagnostic right heart catheterization including right arterial pressure, mean pulmonary arterial pressure, pulmonary vascular resistance, wedge pressure and cardiac output.

Pulmonary hypertension was diagnosed by a mean pulmonary arterial pressure > 25 mmHg and a mean pulmonary capillary wedge pressure <15 mmHg measured at rest. Idiopathic PAH was diagnosed when all other causes of pulmonary hypertension were excluded. Heritable PAH was diagnosed in patients with a known family history of PAH.

**Figure 1:**
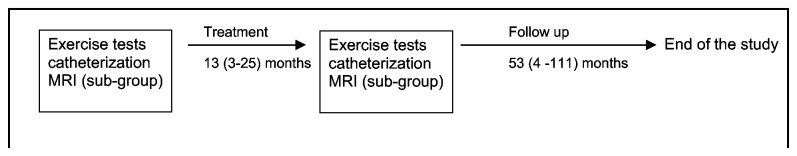
Time schem: First the exercise tests at initial work up, then the second exercise testing after treatment with subsequently the follow up period till the end of the study period.

Patients were treated by a standardized treatment strategy, as reported before [5], and visited the outpatient clinic at least once every three months, according to our institutional protocol. Results from CPET and 6MWD, performed at the time of PAH diagnosis prior i.e to the start of PAH specific treatment were obtained in 65 patients (50 females; mean age 45 ± 2 yrs). In 43 patients (35 females; mean age 44 ± 2 yrs) a second right heart catheterisation and both exercise tests were performed after treatment, 13 (range: 3-25) months after the start of therapy. Cardiac function was measured at baseline by magnetic resonance imaging (MRI) in a subgroup of 34 patients (28 females; mean age 41 ± 3 yrs) of which 19 patients also had MRI data after treatment (Figure 1).

### Cardio pulmonary exercise test

CPET was performed on an electromagnetically braked cycle ergometer (Lode, Groningen, The Netherlands) according to international guidelines [12]. Briefly, three minutes of upright rest were followed by three minutes of unloaded pedalling (0 W) and subsequently a progressive increase in workload (5-20 W min^-1^) to maximum tolerance. The mean exercise test duration was 10 minutes (from unloaded pedaling to peak exercise) in all patients.

All ventilatory variables, calculated from breath by breath measured flow and inspiratory and expiratory gas fractions at the mouth were calculated by a commercial available metabolic computer (V_max__._ 229, CareFusion, Yorba Linda, USA) and analyzed as 20 seconds averages. O_2_pulse was calculated as VO_2_/HR. VE/VCO_2_ was derived from the first linear part of the curve, from unloaded work till the anaerobic threshold [13]. The anaerobic threshold was determined by the combination of the V-slope method and the lowest respiratory equivalent for oxygen [14]. Oxygen saturation of arterial blood (SaO_2_) was determined by pulse-oximetry (9600, Nonin, Plymouth, USA). No patients were using supplemental oxygen during CPET. Heart rate (HR) was measured by electro-cardiography (Eagle 4000, Marquette). Exercise induced shunt was scored using the criteria as described by Sun et al. [15]

### Six minute walking distance

6MWD was measured in all patients as described previously [5] and according to a standardized protocol that follows the American Thoracic Society guidelines.

### Cardiac magnetic resonance imaging

MRI was performed on a Siemens 1.5T Sonata scanner (before April 2008) or on a Siemens 1.5T Avanto scanner (Siemens, Medical Solutions, Erlangen, Germany) equipped with a 6-element phased-array receiver coil. Briefly, stroke volume (SV) was calculated as end-diastolic volume (EDV) minus end-systolic volume and RVEF was calculated as (SV/EDV) * 100% [16].

### Statistical Analysis

Exercise variables included in baseline and change to treatment analyses were 6MWD, maximal VO_2_, HR, O_2_pulse, SaO_2_ and VE/VCO_2_. Absolute exercise variables at baseline and their subsequent changes after the start of treatment were related to survival, which was 63±5 months in the whole group and 53±5 months in the subgroup with available follow-up data. Statistical analysis was performed with the SPSS-15package (SPSS inc, Chicago, USA). All data were expressed as mean values ± standard error of the mean. The unpaired student’s *t* test was used to check for differences between survivors and non-survivors.

Pearson correlations were calculated to check for linear associations between relevant exercise variables (6MWD, VO_2_ and VEVCO_2_slope), pulmonary vascular resistance (PVR; estimated by right hart catheterisation) and RVEF (determined by MRI). Multivariate Cox regression (forward, Wald) analysis was used to identify the independent predictors of survival.

Receiver operating characteristics (ROC) were determined to identify optimal cut-off points for predicting survival based on the highest sum of sensitivity and specificity values. Areas under the ROC curve are presented with a 95% confidence interval (CI). Based on these optimal cut-off points, univariate Kaplan-Meier survival curves were calculated. In all analyses, *p*-values ≤ 0.05 (2-tailed) were considered significant.

## Results

Of the initial group of 65 patients, 19 died and two underwent a lung transplantation (<111 months). Both patients receiving the lung transplant and ten of the deceased patients were included in the subgroup analysis with exercise data after treatment (n=43) (Table S1). For survival analysis patients were followed to a maximum of 111 months after the second set of exercise tests (6MWD,CPET). The mean follow up time was 58 ± 6 months in survivors and 39 ± 8 months in non-survivors.

### Baseline values

**Figure 2:**
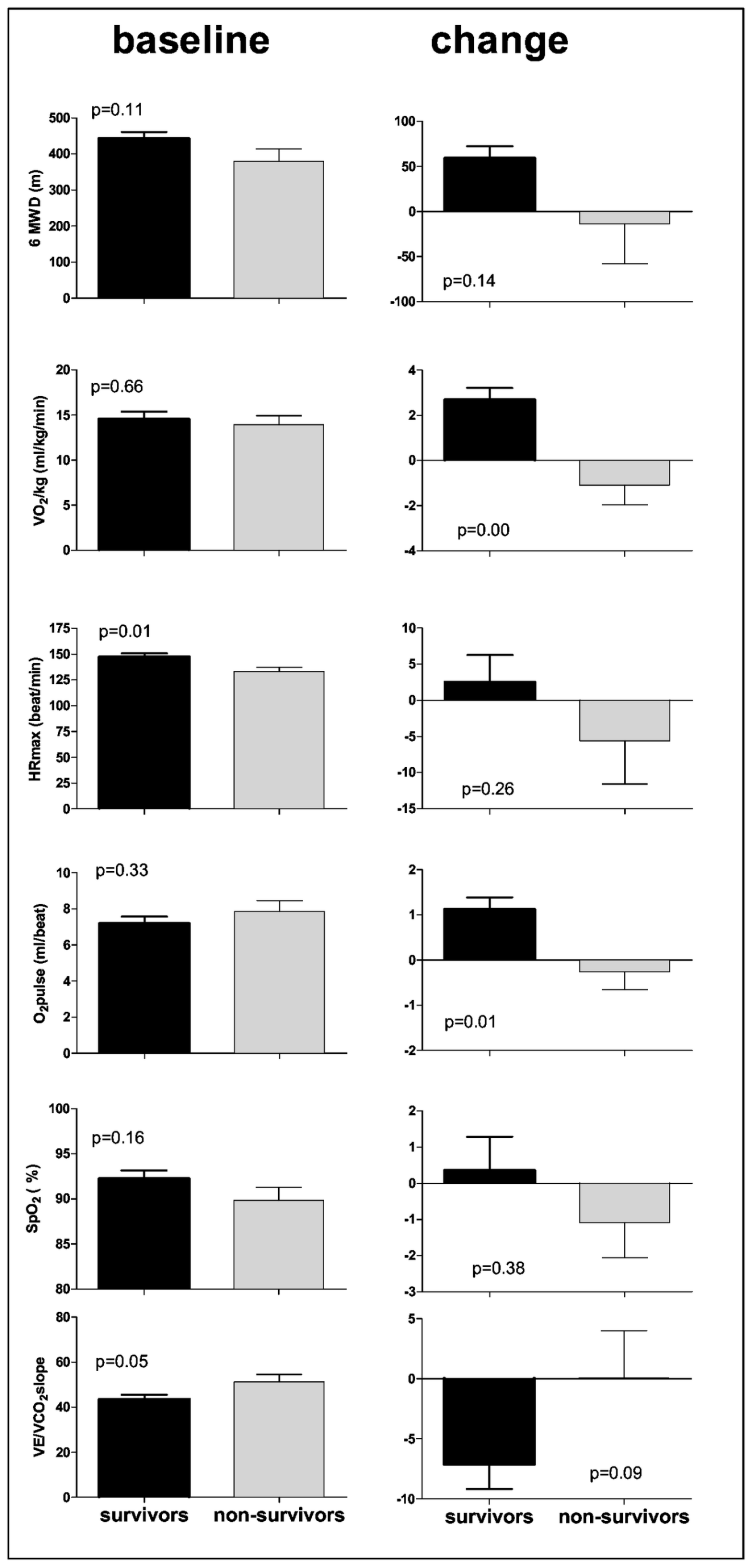
6MWD: six minute walk distance, VO_2_max: peak oxygen uptake, HRmax: peak heart rate, O_2_pulse: Oxygen pulse (=VO _2_max/HRmax), SaO2max: oxygen saturation at maximal exercise, VE/VCO2slope: slope relating ventilation to carbon dioxide production. (mean ± SE) at baseline (n=65) and change over time (n=43).

**Figure 3:**
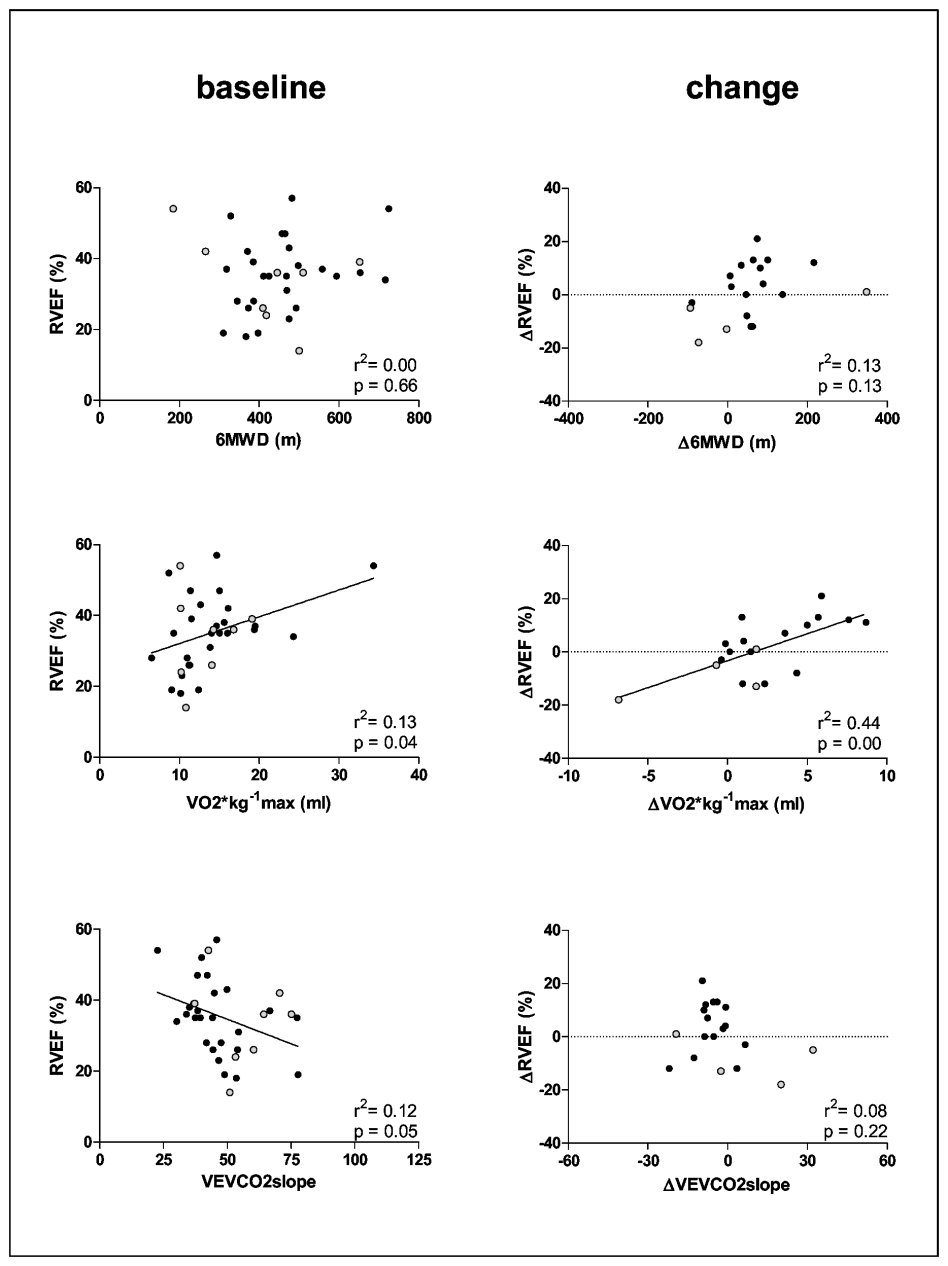
Correlation of exercise variables measured at baseline,(left-panels) as a change from baseline (Δ) (right panels). 6MWD: six minute walk distance, maximum oxygen uptake (VO_2_*kg^-1^) and the slope relating ventilation to carbon dioxide production (VEVCO_2_slope) with right ventricle ejection fraction (RVEF). Open circles = nonsurvivors.

**Figure 4:**
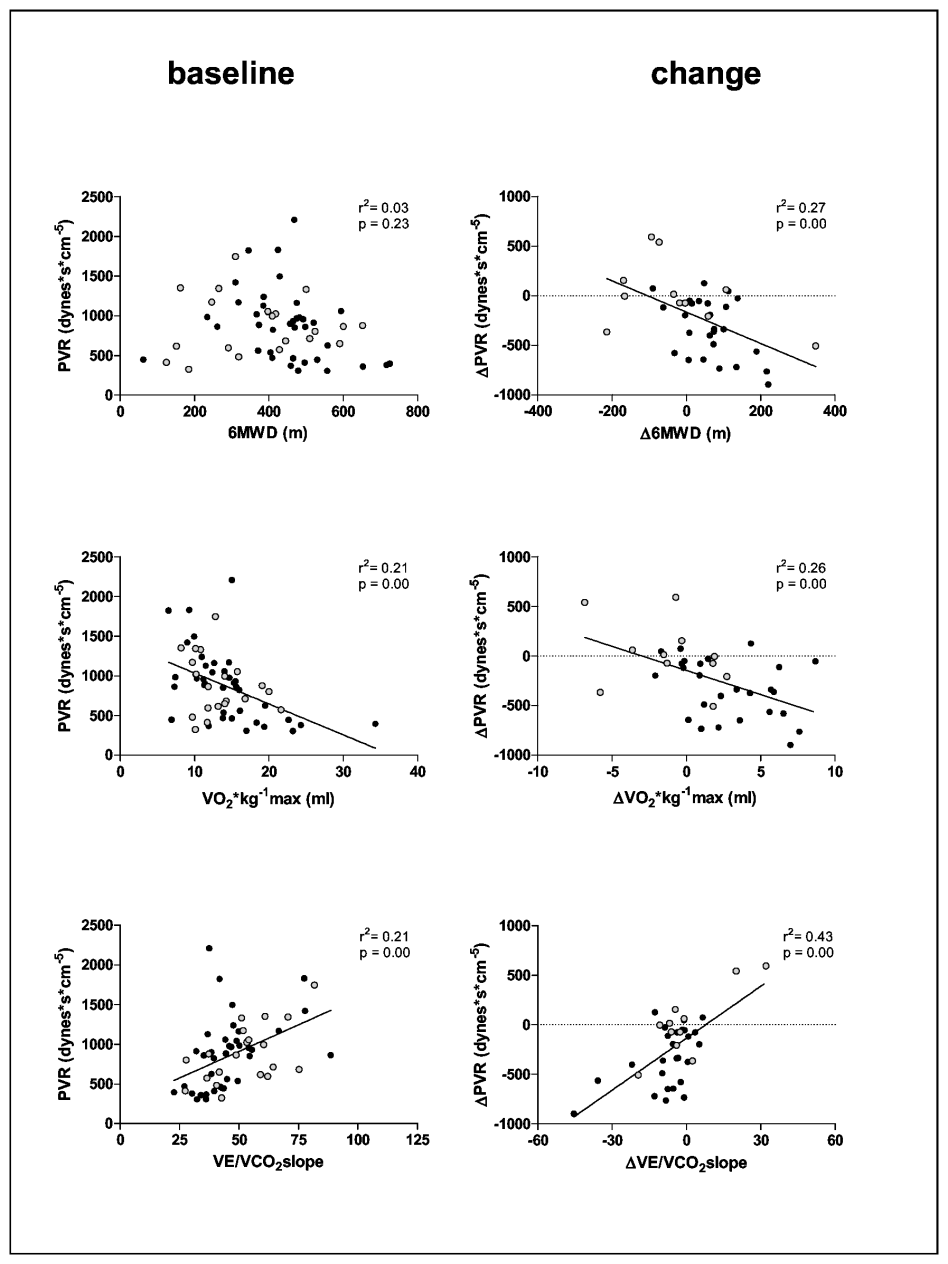
Correlation of exercise variables measured at baseline (left-panels) and as a change from baseline (Δ) (right panels). 6MWD: six minute walk distance, maximum oxygen uptake (VO_2_*kg^-1^) and the slope relating ventilation to carbon dioxide production (VEVCO_2_slope) with pulmonary vascular resistance (PVR). Open circles = nonsurvivors.

As expected in idiopathic PAH, a high percentage of female patients was included. Most demographic and hemodynamic variables at rest were not different between survivors and non-survivors except for venous oxygen saturation (Table 1). Of all baseline exercise variables studied, only maximal HR was significantly higher and the VE/VCO2 slope significantly lower in the survivors (Figure 2, left panels). At baseline, VO_2_max and VEVCO_2_slope correlated significantly with PVR and RVEF. Baseline 6MWD was not associated with PVR or RVEF. Non-survivors were randomly represented in the correlation figures (Figures 3 and 4, left panels). At baseline, two patients showed gas exchange evidence of an exercise induced right to left shunt.

**Table 1:** Baseline demographic and hemodynamic characteristics at time of diagnosis.

	**ALL**	**Survivors**	**Non-Survivors**	***p***
**n**	65	44	21	
**Female (n, %)**	50 (77%)	36 (82%)	14 (67%)	ns
**Age, yr**	45 ± 2	44 ± 2	49 ± 3	0.17
**Height, cm**	167 ± 1	167 ± 1	167 ± 2	0.95
**Weight, kg**	75 ± 2	75 ± 2	76 ± 4	0.77
**Mean PAP, mmHg**	55 ± 2	54 ± 3	56 ± 3	0.73
CO, L*min^-1^	4.7 ± 0.2	4.6 ± 0.2	5.0 ± 0.3	0.36
PVR, dynes*s*cm^-5^	881± 55	883 ± 74	879 ± 83	0.97
**RAP, mmHg**	8.1 ± 0.6	7.9 ± 0.7	8.6 ± 1.0	0.57
**SVO_2_,%**	64 ± 1	66 ± 1	61 ± 2	0.02

PAP: pulmonary arterial pressure, CO: cardiac output, PVR; pulmonary vascular

resistance, RAP; right atrial pressure, SVO_2_: venous oxygen saturation (mean ± SE).

### Changes after treatment

Due to clinical and logistical reasons, 22 out of 65 patients did not perform a second exercise test within 24 months of the initial test. Premature death was not a cause for missing data of a second exercise test. The subgroup with a complete set of exercise tests the variables had identical demographic, hemodynamic and exercise characteristics as the whole initial cohort (see Table S1). After a mean duration of treatment of 13 (range: 3-25) months (Figure 1), survivors showed a significantly greater change in maximal VO_2_ and O_2_pulse than non-survivors. Changes in 6MWD, maximal HR, SaO_2_ and VE/VCO_2_ slope were not significantly different between survivors and non-survivors (Figure 2. right panels).

The exercise induced shunt in two patients remained visible after follow-up. One additional patient showed an exercise induced shunt after follow up.

Changes in VO_2_max upon treatment correlated significantly with both the change in RVEF and the change in PVR. The change in 6MWD and VEVCO_2_slope correlated only with the change in PVR but not with the change in RVEF (Figures 3 and 4). The non-survivors were systematically present in the left lower corner of the ΔVO_2_-ΔRVEF relationship and randomly in the Δ6MWD-ΔRVEF and ΔVE/VCO_2_ slope-ΔRVEF relationship (Figure 3).

### Survival analysis

**Figure 5:**
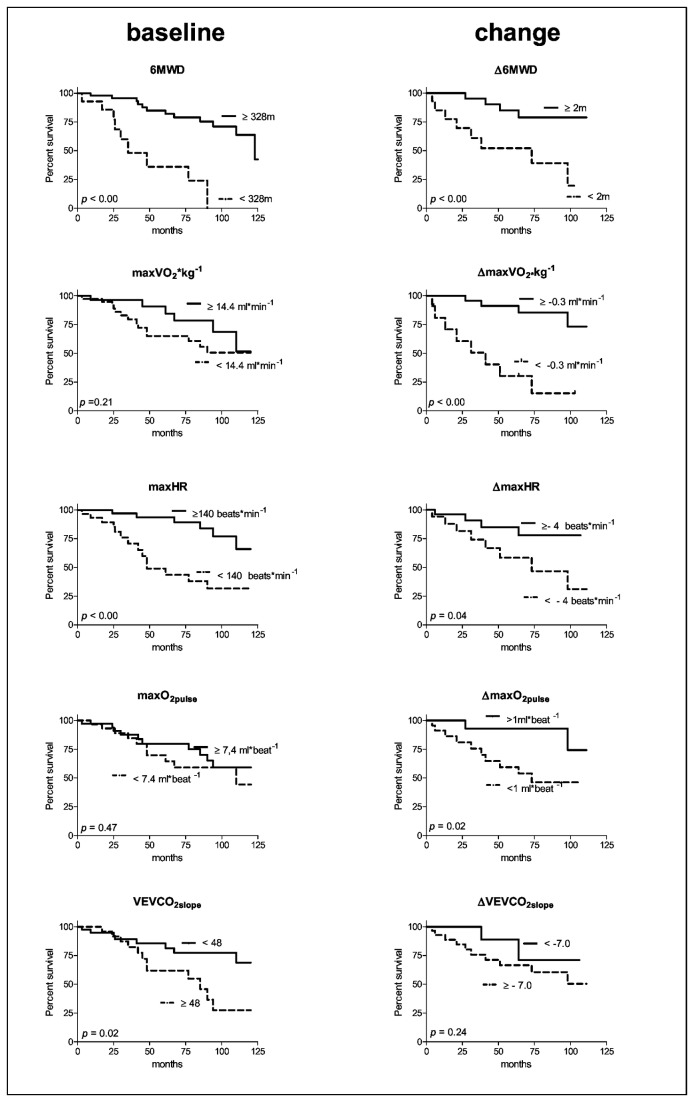
Kaplan-Meier curve’s of baseline exercise variables (left-panels) and changes in exercise variables (Δ) (right panels). 6MWD: six minute walk distance, VO_2_*kg^-1^: oxygen uptake, HR; heart rate, VE/VCO_2 slope_: slope relating ventilation to carbon dioxide production.

The cumulative proportion of survival was 94 ± 3% at 1 yr, 86± 5% at 2 yr, 75 ± 6% at 3 yr, 71 ± 7% at 5 yr, 58 ± 8% at 7 yr and 51 ± 10% at 9 yr. ROC analysis of initial exercise variables identified no significant areas under the curve (Table 2). Baseline 6MWD, maximal HR and VE/VCO_2_ slope were significant predictors of survival as determined by Kaplan-Meier analysis (Figure 5.). Maximal HR was the only baseline independent predictor of survival as identified by multivariate Cox regression (Hazard ratio: 0.97, Wald: 4.21, *p*=0.04).

**Table 2:** Receiver operating characteristics of baseline - and change over time of exercise variables.

		**Area**		**95% CI**		***p***	
**6MWD, m**		0.57		(0.35-0.78)		0.51
**Δ 6MWD, m**		0.71		0.50-0.92)		0.04
VO2max, ml*kg^-1^		0.45		(0.26-0.64)		0.61
ΔVO2max, ml*kg^-1^		0.80		(0.65-0.95)		0.00
HRmax, beats*min ^-^1		0.63		(0.44-0.82)		0.19
Δ**HRmax**, beats*min ^-^1		0.62		(0.43-0.81)		0.23
O2pmax, ml*beat^-1^		0.34		(0.17-0.52)		0.12
ΔO2pmax, ml*beat^-1^		0.76		(0.60-0.92)		0.01
**SaO2max, %**		0.58		(0.37-0.78)		0.44
Δ**SaO2max,%**		0.56		(0.37-0.76)		0.53
**VE/VCO2slope**		0.68		(0.50-0.85)		0.08
**ΔVE/VCO2slope**		0.59		(0.40-0.78)		0.36

CI: confidence interval, 6 MWD: six minute walk distance, VO_2_: oxygen uptake, HR: heart rate, VE/VCO2: slope relating ventilation to carbon dioxide production.

The cumulative proportion of survival of the subgroup of patients with two complete sets of exercise tests was 90 ± 5% at 1 yr, 84± 6% at 2 yr, 77 ± 7% at 3 yr, 73 ± 8% at 4 yr and 69 ± 8% at 5 yr.

**Figure 6:**
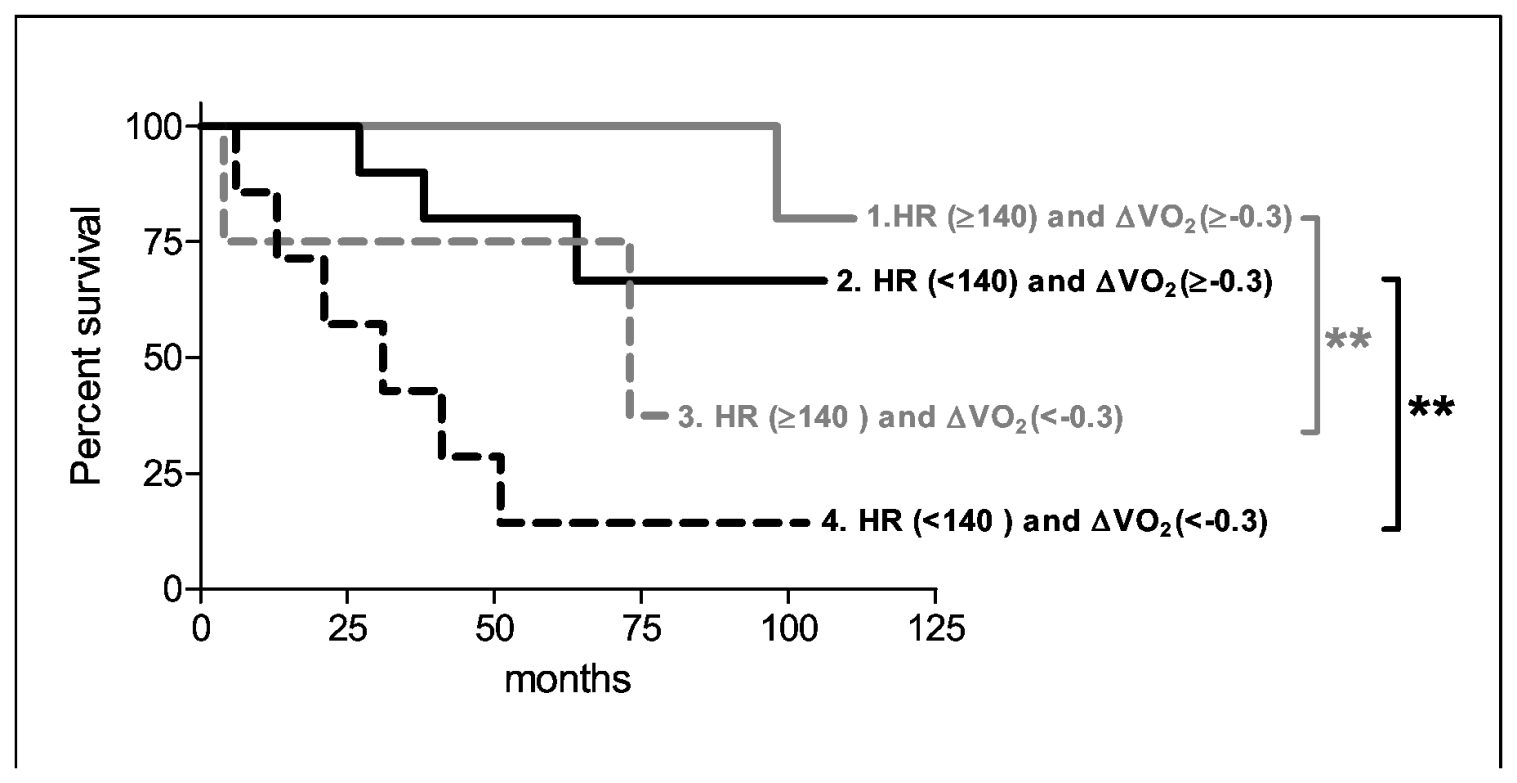
Kaplan- Meier curve of high or low baseline maximal heart rate (HR) in combination with a high or low change of maximal oxygen uptake (ΔVO_2_*kg^-1^) at follow up. ** p < 0.01.

Only the change in 6MWD, maximal VO_2_ and the change in maximal O_2_pulse showed significant areas under the curve by ROC analysis (Table 2.). Changes in 6MWD, maximal VO_2_, HR and O_2_pulse were predictive of survival as determined by Kaplan-Meier analysis (Figure 5.). The change in maximal VO_2_, improved the multivariate Cox regression model when added to baseline maximal HR (Hazard ratio: 0.69, Wald 11.1, *p*=0.001). Figure 6 shows by Kaplan-Meier analysis that regardless of the chronotropic impairment at baseline, a greater improvement in aerobic capacity upon treatment was associated with a better survival.

None of the patients with an exercise induced shunt died during the follow-up period.

Of all included variables only O_2_pulse showed no significant prognostic relevance for survival after treatment (Table 3.)

**Table 3:** Prognostic relevance by Kaplan-Meier analysis of exercise variables determined at baseline and after treatment, and measured as change with treatment.

	**Predictive variable @ baseline**	**Predictive variable after treatment**	**Predictive as change with treatment**
**6MWD**	+	+	+
VO_2_*kg^-1^max		+	+
**HRmax**	+	+	+
**O_2pulse_**			+
**VE/VCO_2slope_**	+	+	

6 MWD: six minute walk distance, VO_2_: oxygen uptake, HR: heart rate, VE/VCO2: slope relating ventilation to carbon dioxide production.

## Discussion

This study shows that in patients with PAH, baseline exercise variables which predict survival are not equally prognostic when measured as changes over time. In this study, next to 6MWD, the chronotropic response and ventilatory efficiency at baseline were significant predictors of long time survival. When analysing changes in exercise variables after an average of 13 months of treatment, changes in 6MWD, maximal aerobic capacity and chronotropic response, but not a change in ventilatory efficiency, were significant predictors of subsequent survival.

### Initial values at time of diagnosis

Survivors and non-survivors were, except for venous saturation, demographically and hemo-dynamically well matched at baseline (Table 1.). At the time of diagnosis, aerobic capacity and ventilatory efficiency correlated significantly with PVR and cardiac function, which is in agreement with earlier research [5,17]. Surprisingly, 6MWD did not show an association with PVR and cardiac function at baseline. Furthermore, we found no significant difference in 6MWD and maximal aerobic capacity between survivors and non-survivors and survival estimated by Kaplan-Meier analysis was not significantly related to aerobic capacity measured during the initial CPET. These findings contrast with earlier research [4,5,17] and although under-powering cannot completely be ruled out, it is likely that these discrepancies between studies are caused by differences in treatment options and patients characteristics, including disease severity. Wensel et al. included more severely affected patients, as reflected by a 20% lower maximal aerobic capacity and a much higher pulmonary vascular resistance [4]. We studied the prognostic value of exercise testing in an earlier study, but then included in addition to patients with idiopathic and heritable PAH, also patients with chronic trombo-embolic pulmonary hypertension [5]. Miyamoto et al. included only idiopathic pulmonary hypertension patients with a more decreased functional capacity as reflected by 30% lower 6MWD compared to the results as reported in our study [17]. We report here in agreement with Wensel et al. [4], that in a group with a less severe exercise intolerance, maximal heart rate during the initial CPET was higher in survivors. Chronotropic incompetence during exercise in severe PAH patients was also recognized by Provencher et al. [18].

An increased ventilatory response for a given degree of CO_2_ production may be caused by over activation of the sympathetic nervous system [19], which by itself is also associated with subsequent mortality in PAH [20]. Hence, the worse prognosis of patients with a decreased ventilatory efficiency at the time of diagnosis is in agreement with earlier and very recent literature [4,5,20,21].

### Changes after treatment

Because a comprehensive CPET is a demanding test for patients and technicians, the relatively simple 6MWD has frequently been used as a clinical end-point [22]. CPET was used in only a few clinical trials to estimate differences in exercise responses upon treatment [23–25]. In the randomized controlled study STRIDE -1 and as part of a composite primary endpoint in a study on the effects of beraprost in PAH, CPET was deemed not useful because no changes in maximal VO_2_ were found after therapy despite improvements in 6MWD [23,25]. This discrepancy between the two estimates of exercise tolerance may probably be due to inexperience and technical difficulties in some of the collaborating exercise laboratories [22].

A clinical study by Oudiz et al. showed significant improvements in aerobic capacity and ventilatory efficiency in treated patients compared to non-treated controls, but the association between changes in CPET variables over time and subsequent survival was not analysed [24].

Although an exercise induced right to left shunt was previously shown to be highly predictive for poor outcome in PAH [11], we could not confirm this finding in our cohort of less severe patients. Only three patients showed a CPET profile corresponding to an exercise induced right to left shunt [15], one of whom only after follow up. None of these patients died during follow up, while the small number of patients with an exercise induced shunt precludes any statistical analysis.

After, on average, 13 months of standard PAH treatment, improvements in 6MWD, maximal aerobic power and maximal O_2_pulse, as well as a smaller deterioration in maximal HR were all predictive of a better survival. A change in aerobic capacity after treatment improved the prognostic value of the model significantly when added to the initially prognostic independent CPET variable maximal HR measured at baseline. An improved aerobic capacity after therapy in surviving PAH patients was also found by McLaughlin et al. after 3 years of therapy [10]. In an early study using CPET and 6MWD to estimate exercise performance after 20 months of infusion with Prostacyclin, VO_2_ and 6MWD improved in a group of 16 primary pulmonary hypertension patients [26]. We could not confirm the data by Provencher et al., who showed that a change in the chronotropic response during exercise after treatment was related to survival [8]. We found no changes in maximal HR or maximal SaO_2_ after therapy, despite improvements in exercise tolerance, which is in agreement with the results of a very early study by Barst et al. [27].

Because changes in maximal HR were similar in survivors and non-survivors, the increased VO_2_ max and O_2_pulse yielded similar results. When maximal HR does not change despite an increase in VO_2_ (= increased O_2_pulse), patients likely improved their stroke volume. An improved stroke volume has been shown to predict survival after PAH specific therapy [8,27].

Despite differences in ventilatory efficiency at the time of diagnosis, changes in VE/VCO_2_ on treatment were not significantly different between subsequent survivors and non-survivors, so that after treatment differences in ventilatory efficiency remain prognostic relevant (Table 3). This is in line with previous observations that PAH specific therapies improve aerobic capacity but not ventilatory efficiency [8–10,22,23,26,27]. It could be speculated that current PAH therapies mainly improve cardio-circulatory function (VO_2_) and do not affect dead space ventilation and/or peripheral chemoreceptor activation. A persistent inefficient ventilation was also shown in patients with left heart failure after heart transplantation [28]. In addition, this study shows that although the change in aerobic capacity and ventilatory efficiency are both associated with a change in PVR, only the change in aerobic capacity is associated with the change in RVEF and has prognostic relevance. These results are explained by the fact that changes in PVR are not always followed by a change in cardiac function which is most important for survival [16].

### Study limitation

A limitation of this study is the relatively small number of patients with available complete sets of exercise - and MRI data at baseline and after treatment. However, it can be judged from the data (Tables S1 and S2) that both subgroups are well representative of the initial study group.

In conclusion, after PAH specific therapy, a change in aerobic capacity reflects an altered cardiac function and has important prognostic relevance. All exercise variables which have prognostic significance when determined at baseline, retain their prognostic relevance after treatment. However, when changes in exercise variables upon treatment are considered, a different set of variables provides prognostic information.

## Supporting Information

Table S1 **Baseline demographic, hemodynamic and exercise characteristics of cohort with exercise data after treatment.** PAP: pulmonary arterial pressure, CO: cardiac output, PVR: pulmonary vascular resistance, RAP: right arterial pressure, SVO_2_: venous oxygen saturation, 6 MWD: six minute walk distance, VO_2_:maximal oxygen uptake, HR; maximal heart rate, SaO_2_: oxygen saturation measured by pulse oximetry, ve/VCO_2_; linear regression slope of ventilation to carbon dioxide production.
(PDF)

Table S2 **Baseline demographic, hemodynamic and exercise characteristics of MRI cohort.** PAP: pulmonary arterial pressure, CO: cardiac output, PVR: pulmonary vascular resistance, RAP: right arterial pressure, SVO_2_: venous oxygen saturation, 6 MWD: six minute walk distance, VO_2_: maximal oxygen uptake, HR; maximal heart rate, SaO_2_: oxygen saturation measured by pulse oximetry, VE/VCO_2_; linear regression slope of ventilation to carbon dioxide production.
(PDF)
