# Supplementary material for: Prognostic Relevance of Changes in Exercise Test Variables in Pulmonary Arterial Hypertension
Source: PLoS One. 2013 Sep 5;8(9):e72013. doi: 10.1371/journal.pone.0072013 (PMC3764059; doi:10.1371/journal.pone.0072013)
Supplement: Table S1 — Baseline demographic, hemodynamic and exercise characteristics of cohort with exercise data after treatment. PAP: pulmonary arterial pressure, CO: cardiac output, PVR: pulmonary vascular resistance, RAP: right arterial pressure, SVO2: venous oxygen saturation, 6 MWD: six minute walk distance, VO2:maximal oxygen uptake, HR; maximal heart rate, SaO2: oxygen saturation measured by pulse oximetry, ve/VCO2; linear regression slope of ventilation to carbon dioxide production. (PDF) [file pone.0072013.s001.pdf]

SUPPLEMENT 1.

Table 4. Baseline demographic, hemodynamic and exercise characteristics of cohort with exercise data after treatment.

	**ALL**	**Survivors**	**Non-Survivors**	***p***
n	43	31	12	
Female (n, %)	35 (81%)	26 (84%)	9 (75%)	> 0.05
Age, yr	44 ± 2	42 ± 3	47 ± 3	0.29
Height, cm	167 ± 1	167 ± 2	167 ± 2	0.94
Weight, kg	75 ± 2	74 ± 2	77 ± 6	0.66
Mean PAP, mmHG	56 ± 3	57 ± 4	52 ± 3	0.45
CO, $L*min^{-1}$	4.5 ± 0.2	4.5 ± 0.2	4.6 ± 0.3	0.68
PVR, $dynes*s*cm^{-5}$	939 ± 65	983 ± 85	832 ± 77	0.30
RAP, mmHg	8.7 ± 0.7	8.5 ± 0.9	9.2 ± 1.5	0.70
SVO_2 ,%	63 ± 1	65 ± 2	60 ± 2	0.13
6 MWD, m	446 ± 17	452 ± 19	429 ± 39	0.56
VO_2, $ml*kg^{-1}$	13.8 ± 0.7	13.7 ± 1.0	14.1 ± 1.1	0.85
HR, $beat*min^{-1}$	144 ± 3	146 ± 4	138 ± 6	0.20
SaO_2, %	91 ± 1	92 ± 1	90 ± 2	0.34
VE/VCO_2	48 ± 2	46 ± 3	54 ± 4	0.12

PAP: pulmonary arterial pressure, CO: cardiac output, PVR: pulmonary vascular resistance, RAP: right arterial pressure, SVO_2: venous oxygen saturation, 6 MWD: six minute walk distance, VO_2: maximal oxygen uptake, HR; maximal heart rate, SaO_2: oxygen saturation measured by pulse oximetry, VE/VCO_2; linear regression slope of ventilation to carbon dioxide production
