# Supplementary material for: Prognostic Relevance of Changes in Exercise Test Variables in Pulmonary Arterial Hypertension
Source: PLoS One. 2013 Sep 5;8(9):e72013. doi: 10.1371/journal.pone.0072013 (PMC3764059; doi:10.1371/journal.pone.0072013)
Supplement: Table S2 — Baseline demographic, hemodynamic and exercise characteristics of MRI cohort. PAP: pulmonary arterial pressure, CO: cardiac output, PVR: pulmonary vascular resistance, RAP: right arterial pressure, SVO2: venous oxygen saturation, 6 MWD: six minute walk distance, VO2: maximal oxygen uptake, HR; maximal heart rate, SaO2: oxygen saturation measured by pulse oximetry, VE/VCO2; linear regression slope of ventilation to carbon dioxide production. (PDF) [file pone.0072013.s002.pdf]

Supplement 2

Table 5. Baseline demographic, hemodynamic and exercise characteristics of MRI cohort.

	**ALL**	**Survivors**	**Non-Survivors**	***p***
n	34	26	8	
Female (n, %)	28 (82%)	21 (81%)	7 (88%)	> 0.05
Age, yr	41 ± 3	41 ± 3	42 ± 4	0.96
Height, cm	167 ± 1	167 ± 2	168 ± 2	0.74
Weight, kg	71 ± 2	72 ± 3	69 ± 5	0.68
Mean PAP, mmHG	59 ± 3	59 ± 4	58 ± 7	0.86
CO, $L*min^{-1}$	4.6 ± 0.2	4.6 ± 0.2	4.9 ± 0.6	0.61
PVR, $dynes*s*cm^{-5}$	980 ± 74	1003 ± 91	910 ± 121	0.60
RAP, mmHg	8.1 ± 0.6	7.9 ± 0.7	8.6 ± 1.2	0.64
SVO_2 ,%	65 ± 1	66± 1	62 ± 3	0.26
6 MWD, m	451 ± 21	459 ± 22	423 ± 52	0.46
VO_2, $ml*kg^{-1}$	13.9 ± 0.9	14.1 ± 1.1	13.2 ± 1.2	0.57
HR, $beat*min^{-1}$	145 ± 3	146 ± 4	145 ± 7	0.89
SaO_2, %	91 ± 1	92 ± 1	88 ± 2	0.10
VE/VCO_2	48 ± 2	46 ± 3	57 ± 5	0.04

PAP: pulmonary arterial pressure, CO: cardiac output, PVR: pulmonary vascular resistance, RAP: right arterial pressure, SVO_2: venous oxygen saturation, 6 MWD: six minute walk distance, VO_2: maximal oxygen uptake, HR; maximal heart rate, SaO_2: oxygen saturation measured by pulse oximetry, VE/VCO_2; linear regression slope of ventilation to carbon dioxide production
